# Interoceptive accuracy correlates with precision of time perception in the millisecond range

**DOI:** 10.3389/fnins.2022.993491

**Published:** 2022-11-14

**Authors:** Maki Uraguchi, Venie Viktoria Rondang Maulina, Hideki Ohira

**Affiliations:** ^1^Department of Psychology, Graduate School of Informatics, Nagoya University, Nagoya, Japan; ^2^Department of Psychology, Atma Jaya Catholic University of Indonesia, Jakarta, Indonesia

**Keywords:** time perception, interoceptive accuracy, vagal activity, temporal generalization task, signal detection theory, sensitivity, precision, accuracy

## Abstract

It has been proposed that accuracy in time perception is related to interoceptive accuracy and vagal activity. However, studies investigating time perception in the supra-second range have provided mixed results, and few studies have investigated the sub-second range. Moreover, there is a lack of studies investigating the relationship between precision in time perception and interoceptive accuracy. A recent meta-analytic review of neuroimaging studies proposed a dynamic interaction between two types of timing processing—an endogenous time keeping mechanism and the use of exogenous temporal cues. Interoceptive accuracy may affect both accuracy and precision of primary temporal representations, as they are generated based on the endogenous time keeping mechanism. Temporal accuracy may vary when adapted to the environmental context. In contrast, temporal precision contains some constant noise, which may maintain the relationship with interoceptive accuracy. Based on these assumptions, we hypothesized that interoceptive accuracy would be associated with temporal precision in the sub-second range, while vagal activity would be associated with temporal accuracy. We used the temporal generalization task, which allowed us to calculate the indices of temporal accuracy and temporal precision in line with the existing research, and also compute the index of participants’ sensitivity according to the signal detection theory. Specifically, we investigated whether (1) interoceptive accuracy would correlate with temporal accuracy, temporal precision, or sensitivity and (2) resting-state vagal activity would correlate with temporal accuracy, temporal precision, or sensitivity. The results indicated that interoceptive accuracy was positively correlated with temporal precision as well as sensitivity, but not with temporal accuracy, in the sub-second range time perception. Vagal activity was negatively correlated only with sensitivity. Furthermore, we found a moderation effect of sensitivity on the relationship between vagal activity and perceived duration, which affected the association between vagal activity and temporal accuracy. These findings suggest the importance of precision as an aspect of time perception, which future studies should further explore in relation to interoception and vagal activity, and of the moderation effects of factors such as participants’ sensitivity in this context.

## Introduction

Accuracy of time perception constitutes the base of adaptive behavior. In daily life, from the circadian rhythm to the range of milliseconds, the perception of time is related to fundamental activities, such as resting, eating, walking, and speaking (Buhusi and Meck, 2005). Nevertheless, the perception of time is modulated or distorted easily by various factors (Matthews and Meck, 2014).

Strong factors of the modulation of time perception are physiological changes, such as body temperature (François, 1927; Hoagland, 1933; Wearden and Penton-Voak, 1995; van Maanen et al., 2019), dopamine level (Maricq and Church, 1983; Meck, 1986; Rammsayer, 1993; Cheng et al., 2006), and pain (Ogden et al., 2015; Rey et al., 2017). The modulation caused by these physiological changes has been explained in relation to the pacemaker-accumulator model of time perception. In this, a pacemaker emits pulses like ticks of a clock. The pulses are accumulated, and the number of accumulated pulses indicates the duration (Creelman, 1962; Treisman, 1963). An increase in body temperature or dopamine level and the existence of pain accelerate the pulse rate of the pacemaker, resulting in the accumulation of more pulses, which leads to subjective lengthening of the duration.

It has been proposed that physiological changes provide the raw material for the feeling of time. According to Craig (2002, 2009), the bodily signals conveyed by the afferent pathways of the autonomic nervous system to the dorsal posterior insula provide the base for the experience of time and a sense of the physiological state of the body. This sense of the physiological state of the body is known as interoception. The bodily signals are processed in the posterior-to-anterior progression in the insula, integrating the inputs from other regions of the brain such as the information of homeostatic, environmental, hedonic, motivational, social, and cognitive factors. Finally, “global emotional moments,” meta-representations of the sentient self, are created in the anterior insular cortex. The accumulation of global emotional moments constitutes the subjective feeling of time. In line with this theory, Wittmann et al. (2010) revealed that the neural activity in the posterior insula increased linearly during the encoding of a temporal task stimulus. This accumulation-like neural activity might signify the use of bodily signals to encode duration. Hashiguchi et al. (2022) found that activity of the right anterior insula was positively correlated with the duration discrimination accuracy. Teghil et al. (2019) performed a meta-analytic review of neuroimaging studies to identify the neural substrates of two types of timing processing: Internally based processing generates the primary temporal representations based on the endogenous time-keeping mechanism independently of the sensory environments, whereas externally cued processing detects the temporal structure of the sensory environment and integrates it with the output of internally based processing to construct temporal representations adapted to the context. Moreover, Teghil et al. (2019) found evidence of a partial dissociation between these timing processes. The insular cortex was found to be activated in internally based processing and more strongly activated in externally cued processing. Therefore, internally based processing may correspond with the use of bodily signals for duration encoding; meanwhile, externally cued processing may reflect the integration of inputs from various brain regions with the primary representation of internally based processing.

If bodily signals are a common base for both time perception and interoception, it can be hypothesized that individuals with higher interoceptive accuracy (Garfinkel et al., 2015), that is, better ability to feel interoception, might display better performance in time estimation. This is because better access to bodily signals might shape the representation of the duration more accurately (Meissner and Wittmann, 2011). However, mixed results have been reported regarding the relationship between interoceptive accuracy measured using the heartbeat counting task (HCT; Schandry, 1981) and the accuracy of the verbal estimation task with stimuli ranging from 19 to 103 s. Some studies have shown a moderate (Craske et al., 2001) or significant (Dunn et al., 2010; Ainley et al., 2014) correlation, while others have reported an insignificant correlation (Ehlers and Breuer, 1992; Zoellner and Craske, 1999; Dunn et al., 2007; Shah et al., 2016a,b; Murphy et al., 2018). Richter and Ibáñez (2021) demonstrated a positive correlation between interoceptive accuracy and estimated seconds of a task lasting 120 s, but not with accuracy in temporal estimation. Furthermore, some studies that used the HCT and duration reproduction task have shown mixed results. For example, although Meissner and Wittmann (2011) demonstrated a positive correlation between interoceptive accuracy and temporal accuracy using 8-, 14-, and 20-s stimuli, Pollatos et al. (2014), who used eight durations of stimuli from 0.5 to 40 s, did not. To our best knowledge, only Cellini et al. (2015) investigated the association between interoceptive accuracy and temporal accuracy in the sub-second range. They used the duration bisection task with 300- to 900-millisecond stimuli and the finger tapping task (free and 1-s paced). Although they did not find a significant association between interoceptive accuracy and temporal accuracy, they detected a positive correlation between vagal activity and temporal accuracy, which was consistent with previous studies (Meissner and Wittmann, 2011; Pollatos et al., 2014) but was followed by opposite results (Fung et al., 2017; Ogden et al., 2019). Moreover, they reported a negative correlation between interoceptive accuracy and the coefficient of variation of inter-tapping intervals in the 1-s tapping task. This indicated that the 1-s temporal production was more precise for individuals with higher interoceptive accuracy.

Both accuracy (less deviation of perceived or estimated duration from the real duration) and precision (less dispersion of the perceived or estimated duration) represent important aspects of timing abilities. Even if mean perceived duration is close to the real duration, timing ability may be poor with high variability in estimates—sometimes very long, sometimes very short—making it less precise (Grondin, 2001, 2010). Nevertheless, the relationship between precision in time perception and interoceptive accuracy has not attracted the interest of researchers.

Precision in time perception comprises two components: the first is proportional variance, which varies with duration, and the other is fixed variance, which is independent of duration (Andrews et al., 2021). It is well known that imprecision (variability) in time perception increases linearly in proportion to perceived duration (Gibbon, 1977; Wearden and Lejeune, 2008). However, this does not hold true for very short durations. This violation of linearity can be explained by assuming some absolute variance independent of duration. If some variance is constant, its relative contribution may be larger in a short duration than in a long duration (Wearden and Bray, 2001). Consistent with this assumption, the Weber fraction in time perception is significantly higher with a noticeably short duration (Getty, 1975; Haigh et al., 2021).

Assuming two types of timing processing, as proposed by Teghil et al. (2019), interoceptive accuracy may be strongly associated with internally based timing processing rather than externally cued timing processing. As internally based processing generates primary temporal representations based on the endogenous time-keeping mechanism, bodily signals are fundamental for it. Therefore, the ability to detect bodily signals may affect the outputs of internally based processing. In contrast, externally cued processing detects the temporal structure in the environment and adapts the primary temporal representations to the context. Therefore, it may have less involvement in bodily signals. Thus, externally cued processing may be less associated with interoceptive accuracy.

We assumed that interoceptive accuracy would reduce the noise (uncertainty) of the primary temporal representations generated through internally based processing, making the representations closer to the real duration at any time. Therefore, both accuracy and precision of the primary temporal representations would be associated with interoceptive accuracy. However, the accuracy of time perception is frequently adapted to the context through externally cued processing. One example is the central tendency known as Vierordt’s law (Vierordt, 1868). A stimulus may be overestimated or underestimated depending on the range of the stimuli set (Jazayeri and Shadlen, 2010). Thus, an association between interoceptive accuracy and the accuracy of perceived duration may not always be observed.

When perceived duration is adapted to the context through externally cued processing, the proportional variance in temporal precision may change with duration; meanwhile, fixed variance may not change. Thus, the association with interoceptive accuracy may be retained only in fixed variance. As the relative contribution of fixed variance is larger in shorter durations, the association between interoceptive accuracy and temporal precision may be observed more prominently in the sub-second range rather than the supra-second range.

Therefore, in the present study, we aimed to investigate the relationship between interoceptive accuracy and time perception in the sub-second range, with the hypothesis that interoceptive accuracy would be positively associated with temporal precision but not with temporal accuracy. We also investigated the relationship between vagal activity and time perception in the sub-second range. As the autonomic nervous system controls homeostasis of the body based on environmental changes, it may be associated with an externally cued timing system. Therefore, we hypothesized that vagal activity would be positively associated with temporal accuracy and expected to replicate the results of Cellini et al. (2015).

For measuring interoceptive accuracy, that is, the ability to feel interoception, we used the HCT (Schandry, 1981). Meissner and Wittmann (2011) and Cellini et al. (2015) measured “interoceptive awareness” and Pollatos et al. (2014) measured “interoceptive sensitivity” with the HCT. Garfinkel et al. (2015) distinguished interoceptive accuracy, which refers to objective accuracy such as performance in the heartbeat detection task, from interoceptive awareness, which is metacognition. Following this, we operationalized interoceptive accuracy with performance in the HCT. Participants were required to detect and count their heartbeats without feeling their pulse using their hands (e.g., without touching their wrist or neck). The difference between the number of counted and real heartbeats was converted into the heartbeat perception score (HPS), which was used as a measure of interoceptive accuracy. Although this task has been used frequently in existing literature, its validity has been questioned due to the possibility of participants estimating their heartbeat using existing knowledge of their heart rate and thus confounding their time estimation ability (Desmedt et al., 2018, 2020). To address this possibility, we explicitly instructed participants to avoid using heart rate knowledge to lessen the confounding effects of non-interoceptive strategies (Desmedt et al., 2018, 2020; Schulz et al., 2021). We also calculated the correlation between the number of counted heartbeats and the estimated duration in the temporal ability task to determine whether participants used their ability of time perception in the HCT (Desmedt et al., 2020).

For measuring temporal abilities, we used the temporal generalization task (Church and Gibbon, 1982; Wearden, 1992), which has been used in many studies on time perception in the sub-second range. We set two conditions in the task difficulty based on the study by Ferrara et al. (1997) to manipulate the involvement of exogenous factors and compared the results. In the temporal generalization task, participants first memorized the standard duration and then judged whether the seven comparison stimuli had the same duration as the standard. The stimuli that received the response “same” indicated the duration of the subjective standard in memory. We calculated the weighted mean of the subjective standard (MSS) as a measure of perceived duration. Then, in line with previous research, we calculated the absolute difference (AD) between MSS and real standard as a measure of temporal accuracy, and dispersion of the subjective standard (DSS) as a measure of temporal precision. However, according to the signal detection theory (Green and Swets, 1966), the results of the temporal generalization task confounded participants’ sensitivity and their criterion about the responses (Blough, 1967; Wearden, 2008). Thus, we also calculated the indicator of sensitivity *A’* (Pollack and Norman, 1964; Macmillan and Creelman, 1996). *A’* was less affected by the exogenous factors, as it was independent of the response criterion.

In sum, we investigated whether (1) interoceptive accuracy explained AD (temporal accuracy), DSS (temporal precision), or *A’* (sensitivity independent of response criterion) and (2) vagal activity explained AD, DSS, or *A’*. Additionally, we investigated the moderation effect of interoceptive accuracy on the relationship between DSS and vagal activity. We also investigated the moderation effects of interoceptive accuracy, DSS, and *A’* on the relationship between MSS (perceived duration) and vagal activity to determine possible effects of individual differences related to the internally based timing system.

## Materials and methods

### Participants

Thirty-four healthy undergraduate and graduate students participated in the experiment (16 women, age 18–24 years, mean age 21.06 years). All participants provided written informed consent before the experiment began. No participant reported difficulties in hearing. We obtained data from 32 participants, excluding data for two that could not be recorded due to problems in the equipment.

### Experimental design

A within-subject design was used. All participants took part in two conditions of the experiment—easy and difficult—which used stimuli spaced 150 and 75 milliseconds, respectively. The order of the conditions was counterbalanced.

### Procedure

After providing informed consent, participants entered a shield room. Disposable electrodes were attached on their forearms. A pulse wave was recorded during the resting period of 10 min. Then, participants performed the HCT, which was followed by the temporal generalization task. Before each task, brief instructions were given and participants had time to practice and ask questions if they were uncertain about how to perform the task. The present study was approved by the Ethics Committee of Nagoya University (approval no. NUPSY-180914-G-01).

#### Resting state heart rate recording

During a resting period of 10 min, the participants’ pulse waves were measured. Participants sat on a chair with their arms extended on a table in front of them and their palms facing upward. They were instructed not to move their body, to feel relaxed, to keep their eyes open except when blinking, not to sleep, and to breath naturally. The pulse wave was recorded with Biopac MP150 system (Biopac Systems, Inc., Goleta, CA, USA).

#### Heartbeat counting task

In the HCT, participants were instructed to detect and count their heartbeats without feeling their pulse using their hands (e.g., without touching their wrist or neck) and report the number of heartbeats they detected during periods of 25, 35, and 45 s. Meanwhile, their pulse waves were recorded. Participants were seated quietly in a chair in a position in which their limbs did not touch each other or the body, and their back did not touch the backrest of the chair. Their arms were extended on a table in front of them. The cues “Start” and “Stop,” which indicated the periods to count heartbeats, were displayed on a CRT monitor. No participant reported any difficulties in recognizing the cues. After recording the resting state heart rate for 10 min in a quiet shield room, participants were given the following instructions: “You will count your heartbeats several times. You will not be informed in advance the duration of each interval for counting,” “Do not use the knowledge such as the average heart rate of humans for guessing,” “Do not take your pulse or attempt to use any manipulation that facilitates the heartbeat counting except hearing your heartbeats,” “If it is difficult to sense your heartbeats, count the rhythm that you assume similar to your heartbeats,” and “Please try your best to sense your heartbeats.”

#### Temporal generalization task

The stimuli were 500 Hz pure tones. The duration of the standard stimulus was 600 milliseconds and for the comparison stimuli were 150, 300, 450, 600, 750, 900, and 1050 milliseconds in the easy condition and 375, 450, 525, 600, 675, 750, and 825 milliseconds in the difficult condition. All experimental events were controlled by PsychoPy2 application (Peirce, 2007, 2009; Peirce et al., 2019). An LCD monitor displayed the instructions and cues. Participants listened to the stimuli through speakers that were placed on the right and left sides of the monitor. A computer keyboard registered the participants’ responses.

The experiment consisted of 42 sets. Each set began with a presentation of the standard stimuli, followed by seven trials with the comparison stimuli. Each comparison stimulus was presented once in a set in a pseudorandom order.

Participants were instructed that when a cross was displayed on the monitor, they should listen to the sound and memorize the duration, and when a question mark was displayed, they needed to judge whether the sound had the same duration as the memorized one, then report by key pressing.

The left and right arrow keys were used to indicate responses of “same” and “not same.” The direction of the keys was counterbalanced. No feedback to responses was given.

At the beginning of a set, a cross appeared on the monitor. Then, the standard was presented five times continuously with interstimulus intervals of 1.5 s. After the presentation of the standard, the cross disappeared and a question mark was displayed immediately. The first comparison was presented 1.7 s after the onset of the question mark. After listening to the comparison, participants pressed one of the designated keys to report whether the comparison was of the same duration as the standard. The next comparison was presented 1.7 s after the key-press.

### Data processing

#### Resting state heart rate variability

Root mean square of successive differences (RMSSD) of RR intervals and high frequency (HF) component were obtained as the indicators of vagal activity by a short-term (at least 5-min) heart rate variability (HRV) analysis for each participant, following the standard method (Malik, 1996; Min et al., 2008). Considering the time required for participants to adapt to the environment, the data of the latter part of the recording period were used. RR intervals were calculated from the pulse wave data by the software AcqKnowledge 4.2 (Biopac Systems, Inc, Goleta, CA, USA), then processed by a freeware, Kubios HRV Standard.

#### Heartbeat perception score

Interoceptive accuracy was quantified by the heartbeat perception score (HPS) according to Schandry (1981). The number of objective heartbeats was obtained from the number of R-waves in the corresponding periods. The error score was calculated as the difference in the absolute value of the number of times the heartbeats were subjectively counted and objectively measured in a corresponding period—25, 35, or 45 s—normalized by the number of times the heartbeats were objectively measured. Finally, the error score was deducted from one to obtain the HPS. For the statistical analysis, the mean of HPS in three measurements was used. A higher HPS indicated higher interoceptive accuracy.


$$\mathrm{HPS}=1-\frac{\begin{array}{c} |\mathrm{subjectively}\mathrm{counted}\mathrm{heartbeat}\\ -\mathrm{objectively}\mathrm{measured}\mathrm{heartbeat}| \end{array}}{\begin{array}{c} \mathrm{objectively}\,\mathrm{measured}\,\mathrm{heartbeat} \end{array}}$$

#### Indices from the results of the temporal generalization task

The results of the task were depicted as the temporal generalization gradient for the easy and difficult conditions separately. The proportion of “same” responses in each comparison stimulus was calculated and plotted against each corresponding comparison stimulus.

Mean of the subjective standard (MSS) was calculated as a weighted mean of all comparison stimuli to which a “same” response had been obtained. We used the Equation I of Klapproth and Wearden (2011). They used the equation to examine the shift of the temporal generalization gradients numerically. The sum of the products of each stimulus (*duration* in the following equation) and frequency of “same” responses to the corresponding stimulus (*b* in the following equation) was divided by the sum of the frequency of all “same” responses.


$$MSS\;=\frac{\begin{array}{l} b_{1}\times duration_{1}+b_{2}\times duration_{2}\\ \;\;\;\;\;+......+b_{n}\times duration_{n} \end{array}}{b_{1}+b_{2}+.............+b_{n}}$$

Absolute difference (AD) was calculated as follows using MSS. A smaller AD indicated higher temporal accuracy.


A⁢D=|M⁢S⁢S-600|

Dispersion of the subjective standard (DSS) was calculated using Equation II of Klapproth and Wearden (2011). They used the equation to examine the steepness of the temporal generalization gradients, which indicated how precisely participants judged durations in a temporal generalization task. The square root of the summation of the squared differences between each stimulus (*duration*) and mean subjective standard (*MSS*) weighted by the frequency of “same” responses to the corresponding stimulus (*b*) was divided by the summation of the frequency of “same” responses. A smaller DSS indicated higher temporal precision in task performance.


$$D\,S\,S=\frac{\sqrt{\begin{array}{c} b_{1}\times {\left(d\,u\,r\,a\,t\,i\,o\,n_{1}-M\,S\,S\right)}^{2}+b_{2}\times {\left(d\,u\,r\,a\,t\,i\,o\,n_{2}-M\,S\,S\right)}^{2}\\ +\cdots \,\cdots \,b_{n}\times {\left(d\,u\,r\,a\,t\,i\,o\,n_{n}-M\,S\,S\right)}^{2} \end{array}}}{b_{1}+b_{2}+\cdots \,\cdots +b_{n}}$$

*A’* was calculated according to Grier (1971). Both *d’* and *A’* are the indices of sensitivity independent of the response criterion in the theory of signal detection. While *d’* indicates the distance between the means of the hypothesized distributions, *A’* indicates the area under the iso-sensitivity (receiver operating characteristic) curve. *A’* can be used without the assumption of equal variance and is reportedly more accurate than *d’* when the variances of hypothetical distributions are not equal (Donaldson, 1993). We used *A’* instead of *d’* because it was not possible in our experiment to obtain data to evaluate the equal variance of the distributions. The responses of 294 trials were categorized into four groups according to the signal detection theory: (1) “same” when the real standard values were hit; (2) “same” when the non-standard values were false alarms; (3) “not same” when real standard values were missed; and (4) “not same” when the non-standard values were correct rejections. The hit rate (*y* in the following equation) and false-alarm rate (*x* in the following equation) were calculated. The value of hit rate minus false-alarm rate was multiplied by the same value added to one. This product was divided by the product of the hit rate multiplied by four and one minus the false-alarm rate. Finally, the quotient was added to 0.5. Although the minimum value of *A’* was zero, an *A’* less than 0.5 might signify sampling error or confusion in response. A higher *A’* indicated higher sensitivity without the influence of the response criterion (Stanislaw and Todorov, 1999).


$$A^{\prime }=\frac{1}{2}+\frac{\left(y-x\right)\,\left(1+y-x\right)}{4\,y\,\left(1-x\right)}$$

### Statistical analyses

The statistical analyses were performed using R version 3.6.3 (R Core Team, 2020). For the linear regression model analysis, the car package (Fox et al., 2012) was used. Models with the interaction term were tested using analysis of variance (ANOVA)–type III.

#### Data distribution

The normality of the data was verified using Shapiro–Wilk test. The histograms, box plots, and violin plots were checked visually. HPS, MSS, DSS, and *A’* were distributed normally. As the RMSSD and HF component did not distribute normally, data were transformed with a natural logarithm. The AD showed a zero-truncated distribution. As we recognized it as its nature, log transformation was not applied. To avoid skewing due to outliers, data for three participants whose AD, MSS, or DSS showed extreme values, confirmed by Smirnov–Grabbs test (*p* < 0.050), were omitted. Thus, statistical analyses were performed with the data of 29 participants. The distribution of HPS of the 29 participants compared with a previous study. The average number of counted heartbeats for a minute was not distributed normally.

#### Comparison between the easy and difficult conditions

Significant differences and similarities of the data between the easy and difficult conditions were verified. The proportions of the “same” response to the physically identical stimuli under easy and difficult conditions, 450, 600, and 750 milliseconds, were examined using a paired *t*-test. Regarding AD, the exact Wilcoxon signed rank test and Spearman’s correlation analysis were used. Regarding MSS, DSS, and *A’*, a paired *t*-test and Pearson’s correlation analysis were used.

#### Correlations between variables

Correlations between (1) RMSSD and HF component, (2) HPS (interoceptive accuracy) and vagal activity, (3) HPS and AD (temporal accuracy), DSS (temporal precision), *A’*(sensitivity), and (4) vagal activity and AD, DSS, *A’* were verified. The correlations between AD and other variables were verified using Spearman’s correlation analysis. Correlations between the variables, which were normally distributed, were investigated using Pearson’s correlation analysis.

Correlation between the average number of counted heartbeats for a minute and the MSS was verified using Spearman’s correlation analysis.

#### Interaction between variables

The moderation effect of interoceptive accuracy on the relationship between DSS and vagal activity was investigated using the model with DSS as the dependent variable and RMSSD (or HF component) and interoceptive accuracy as the independent variables with the interaction term, assuming that a more variable heart rate would lead to more variable DSS, and this association would be more prominent with higher interoceptive accuracy.

Thereafter, the moderation effects of interoceptive accuracy, precision, and sensitivity on the relationship between MSS and vagal activity were investigated, assuming that individual differences related to the internally based timing system would make a difference in the perceived duration under the same vagal activity. The linear regression models with interaction term were tested. The models with MSS as the dependent variable were tested with the following independent variables: (1) RMSSD and HPS with the interaction term, (2) HF component and HPS with the interaction term, (3) RMSSD and DSS with the interaction term, (4) HF component and DSS with the interaction term, (5) RMSSD and *A’* with the interaction term, and (6) HF component and *A’* with the interaction term. Hierarchical linear regression analysis was conducted to confirm the effect of the interaction term. The data used in the modeling were mean-centered, which did not affect the results (Shieh, 2011). The variance inflation factor (VIF) was calculated for each factor to verify the multicollinearity.

## Results

### Temporal generalization gradients

**FIGURE 1:**
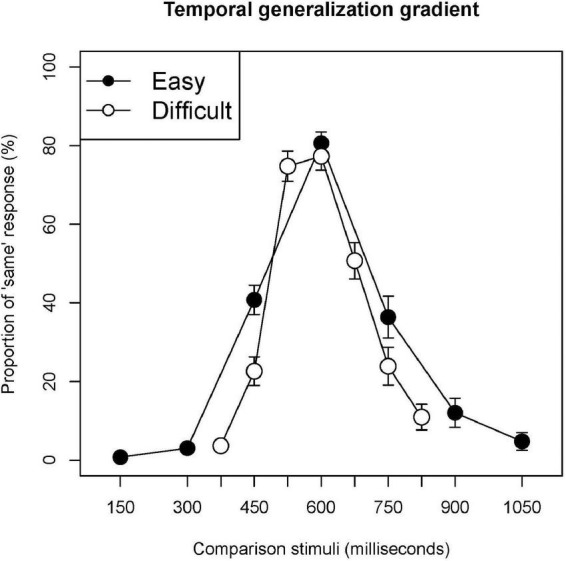
Temporal generalization gradients. Filled and open circles indicate the proportion of “same” responses corresponding to each stimulus under the easy and difficult conditions, respectively. Error bars are standard error.

Figure 1 shows the temporal generalization gradients obtained under the easy and difficult conditions. The location of the peak was at the standard stimulus under both conditions. Under the difficult condition, the proportion of “same” responses to the stimulus of 525 millisecond was as high as the peak. The gradient in the difficult condition was steeper than that in the easy condition.

The proportion of “same” responses under the difficult condition was significantly lower than that under the easy condition for 450 and 750 milliseconds (*t* = 5.499, *p* = 0.000; *t* = 4.694, *p* = 0.000, respectively). The proportion of “same” responses for 600 milliseconds did not differ significantly between the conditions (*t* = 1.727, *p* = 0.095).

### Heartbeat perception score

**FIGURE 2:**
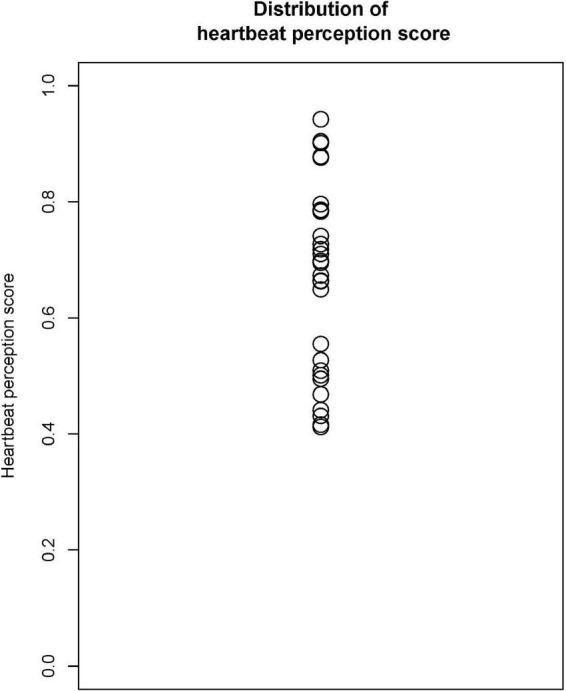
Distribution of the heartbeat perception score (HPS). Mean = 0.667, *SD* = 0.162.

Figure 2 shows the distribution of the HPS (mean = 0.667, *SD* = 0.162).

Table 1 shows the counted and recorded numbers of the heartbeats in the periods of 25, 35, and 45 s, with the average heartbeats per minute.

**TABLE 1:** Mean of the counted and recorded heartbeats and the average for a minute.

	25 s	35 s	45 s	Average beats for a minute
Counted heartbeats	21.655	29.241	36.172	50.110
Recorded heartbeats	30.379	42.552	55.379	73.232

The correlation between the average number of heartbeats counted per minute and the MSS was not significant under the easy condition (rho = −0.146, *p* = 0.447), nor under the difficult condition (rho = −0.345, *p* = 0.067).

### Comparison between the easy and the difficult conditions

**FIGURE 3:**
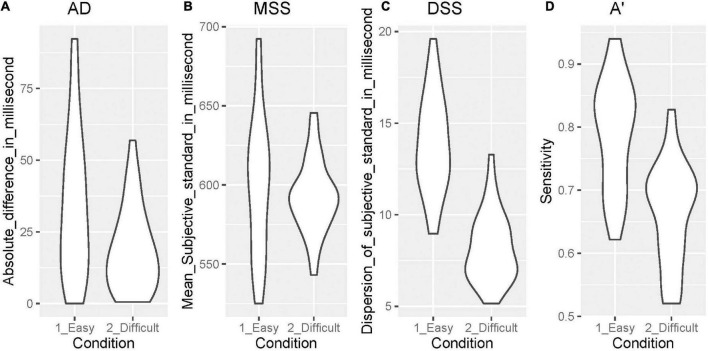
Data distribution of AD, MSS, DSS, and *A’*. **(A)** AD, **(B)** MSS, **(C)** DSS, **(D)** *A’*. AD, absolute difference between the real standard and mean of the subjective standard; MSS, mean of the subjective standard; DSS, dispersion of the subjective standard; A, sensitivity independent of the response criterion according to the signal detection theory.

Figure 3 shows the data distribution of AD (temporal accuracy), MSS (perceived duration), DSS (temporal precision), and *A’* (sensitivity to the duration independent of the response criterion), comparing the easy and difficult conditions.

We found a significant difference in the AD (*V* = 36, *p* = 0.001) between the easy (median = 30.000, 1st–3rd quartile = 16.406–48.214) and difficult conditions (median = 15.203, 1st–3rd quartile = 6.034–26.124). There was also a significant difference in the DSS (*t* = 15.616, *p* = 0.000) between the easy (mean = 13.850, *SD* = 2.657) and difficult conditions (mean = 8.196, *SD* = 1.884). Further, there was a significant difference in *A’* (*t* = 13.416, *p* = 0.000) between the easy (mean = 0.791, *SD* = 0.086) and difficult conditions (mean = 0.664, *SD* = 0.080). There was not a significant difference only in the MSS (*t* = 1.727, *p* = 0.095) between the easy (mean = 600.142, *SD* = 43.758) and difficult conditions (mean = 592.428, *SD* = 23.047).

**FIGURE 4:**
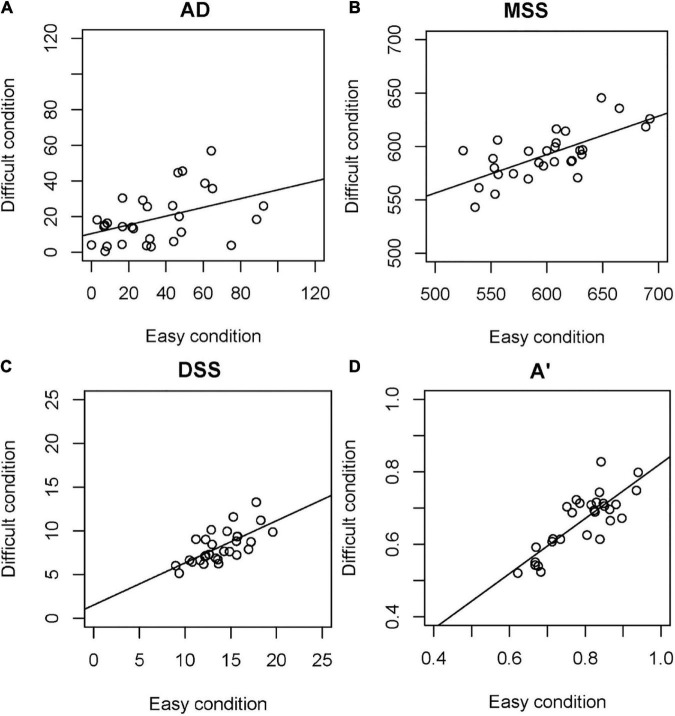
Correlation between easy and difficult conditions. **(A)** AD, **(B)** MSS, **(C)** DSS, **(D)** *A’*. AD, absolute difference between the real standard and mean of the subjective standard; MSS, mean of the subjective standard; DSS, dispersion of the subjective standard; *A’*, sensitivity independent of the response criterion according to the signal detection theory.

Figure 4 shows the correlations between the easy and difficult conditions. With all time perception variables, the correlations between the easy and difficult conditions were significant: AD (rho = 0.424, *p* = 0.022); MSS (*r* = 0.684, *p* = 0.000); DSS (*r* = 0.680, *p* = 0.000); and *A’* (*r* = 0.816, *p* = 0.000).

### Correlation analysis between variables

Table 2 shows the results of the single correlation analysis.

**TABLE 2:** Correlations between interoceptive accuracy, vagal activity, and indices of temporal task performance.

	Easy			Difficult				
		
	AD	DSS	*A’*	AD	DSS	*A’*	RMSSD	HF
HPS	–0.084	−0.401*	0.506**	–0.015	–0.169	0.274	–0.194	0.011
RMSSD	0.328†	0.130	−0.342†	0.064	0.170	−0.384*	–	0.919***
HF	0.294	0.173	−0.385*	0.020	0.075	−0.345†	0.919***	−

†p < 0.010, *p <0.050, **p < 0.010, and ***p < 0.001. The coefficients of the relation with AD are indicated in rho obtained by Spearman’s correlation analysis. Other coefficients are described in r obtained by Pearson’s correlation analysis. HPS, heartbeat perception score; RMSSD, root mean square of successive differences of RR intervals; HF, high frequency component of heart rate variability; AD, absolute difference between the real standard and the mean of the subjective standard; DSS, dispersion of the subjective standard; *A*’, sensitivity independent of the response criterion according to the signal detection theory.

The indicators of vagal activity were highly correlated to each other (*r* = 0.919, *p* = 0.000), while neither RMSSD nor HF component correlated significantly with HPS, interoceptive accuracy (*r* = −0.194, *p* = 0.314; *r* = 0.011, *p* = 0.955, respectively).

Dispersion of the subjective standard, precision in time perception, correlated significantly with HPS, interoceptive accuracy, under the easy condition (*r* = −0.401, *p* = 0.031) but not under the difficult condition (*r* = −0.169, *p* = 0.381). The sensitivity, *A’*, also correlated significantly with HPS under the easy condition (*r* = 0.506, *p* = 0.005) but not under the difficult condition (*r* = 0.274, *p* = 0.150). Meanwhile, AD, accuracy in time perception, did not correlate with HPS under any condition.

Regarding the relationship with vagal activity, only *A’* correlated significantly with RMSSD under the difficult condition (*r* = −0.384, *p* = 0.040) but not under the easy condition (*r* = −0.342, *p* = 0.069). *A’* also correlated significantly with HF component only under the easy condition (*r* = −0.385, *p* = 0.039) but not under the difficult condition (*r* = −0.345, *p* = 0.067). Neither AD nor DSS had a significant association with vagal activity.

### Interactions effects between heartbeat perception score, vagal activity, precision, and sensitivity

The VIF of all models was less than 2.0.

Testing the models with the interaction term, with DSS as the dependent variable and RMSSD (or HF component) and HPS as the independent variables, the interaction term was not significant under any condition: RMSSD and HPS under the easy condition (*p* = 0.846), under the difficult condition (*p* = 0.612); HF component and HPS under the easy condition (*p* = 0.686), under the difficult condition (*p* = 0.529).

**FIGURE 5:**
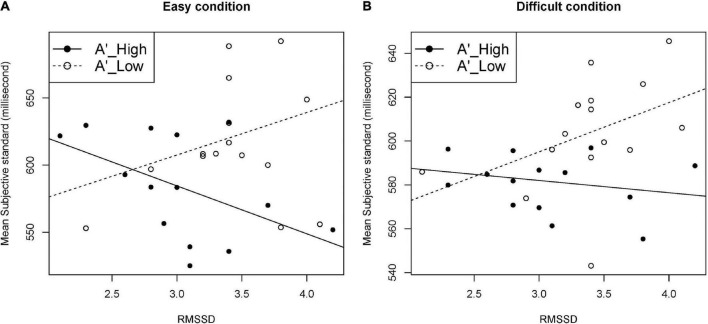
Moderation effect of *A’* on the relationship between RMSSD and MSS. Data were split by median of *A’* into higher-*A’* group (*n* = 14) and lower-*A’* group (*n* = 15). **(A)** Easy condition, **(B)** difficult condition. *A’*, sensitivity independent of the response criterion according to the signal detection theory; RMSSD, root mean square of successive differences of RR intervals; MSS, mean of the subjective standard.

Testing six models with the interaction term with MSS as the dependent variable, only the model with *A’* and RMSSD as independent variables showed a significant interaction effect under easy and difficult conditions (*p* = 0.030, and *p* = 0.038, respectively). The effect of the interaction term was confirmed also by the hierarchical linear regression analysis under easy and difficult conditions (*p* = 0.030, and *p* = 0.038, respectively). Table 3 shows the models and results of the hierarchical linear regression analysis. Table 4 shows the summary of the models with the interaction term. Figure 5 shows the moderation effect of *A’* on the relationship between RMSSD and MSS.

**TABLE 3:** Hierarchical linear regression analysis.

Model 1	*MSS* = *b*_0_ + *b*_1_×*A*′ + *b*_2_×*RMSSD* + ε				
Model 2	*MSS* = *b*_0_ + *b*_1_×*A*′ + *b*_2_×*RMSSD* + *b*_3_×*A*′×*RMSSD* + ε				

	*R* ^2^	RSS	Sum of Sq	*F*	*p*
**(A) Easy condition**					
Model 1	0.387	32885			
Model 2	0.494	27131	5753	5.301	0.030
**(B) Difficult condition**					
Model 1	0.388	9099.2			
Model 2	0.487	7630.3	1468.9	4.8128	0.038

(A) Easy condition, (B) difficult condition. *A*’, sensitivity independent of the response criterion according to the signal detection theory; RMSSD, root mean square of successive differences of RR intervals.

**TABLE 4:** Summary of models with the interaction term.

.(A) Easy condition				

	b	SE of b	*t*	*p*
Intercept	593.425	6.777	87.560	0.000
*A’*	–252.943	84.902	–2.979	0.006
RMSSD	–4.781	13.482	–0.355	0.726
*A’* × RMSSD	–446.140	193.766	–2.302	0.030

**(B) Difficult condition**				

	**b**	**SE of b**	***t***	***p***

Intercept	588.394	3.729	157.782	0.000
*A’*	–146.277	45.857	–3.190	0.004
RMSSD	9.908	7.474	1.326	0.197
*A’* × RMSSD	–256.078	116.728	–2.194	0.038

(A) Easy condition, (B) difficult condition. *A*’, sensitivity independent of the response criterion according to the signal detection theory; RMSSD, root mean square of successive differences of RR intervals.

The interaction terms of the other five models were not significant under any condition: RMSSD and HPS (*p* = 0.055, *p* = 0.068, respectively); HF component and HPS (*p* = 0.083, *p* = 0.207, respectively); RMSSD and DSS (*p* = 0.050, *p* = 0.822, respectively); HF component and DSS (*p* = 0.068, *p* = 0.737, respectively); and HF component and *A’* (*p* = 0.050, *p* = 0.088, respectively).

## Discussion

We investigated the relationship between time perception in the sub-second range and interoceptive accuracy, with the hypothesis that interoceptive accuracy would be associated with precision, not with accuracy, in temporal task performance. AD (temporal accuracy) did not correlate with interoceptive accuracy. Furthermore, DSS (temporal precision) and *A’* (sensitivity independent of the response criterion) correlated significantly with interoceptive accuracy. Higher interoceptive accuracy was associated with higher precision as well as higher sensitivity. Therefore, the hypothesis was supported. We also investigated the relationship between vagal activity and time perception in the sub-second range, hypothesizing that vagal activity would be associated with temporal accuracy. However, AD did not have a significant association with vagal activity. Therefore, the hypothesis was not supported.

A significant correlation between interoceptive accuracy and temporal precision was observed only under the easy condition. The difficulty in the task resulted in a steeper temporal generalization gradient with a significant decrease of the “same” response proportion, replicating the results of the study by Ferrara et al. (1997). Naturally, the DSS of the difficult condition was lower than that of the easy condition, indicating more precision. Nevertheless, according to Ferrara et al. (1997) and Wearden (2008), this difference was caused by the change in the participants’ response threshold or criterion, not sensitivity. Namely, the difficulty of the task made participants more conservative by responding with “same.” Indeed, our results showed higher sensitivity *A’* under the easy condition. We hypothesized the association between interoceptive accuracy and temporal precision assuming that the noise of the primary temporal representation, which is associated with interoceptive accuracy, may be retained only in the fixed variance, and that the relationship between interoceptive accuracy and temporal precision may be prominent in the sub-second range because the relative contribution of the fixed variance in temporal precision might be larger in shorter durations. In the difficult condition, temporal precision was more affected by the response criterion. Hence, the relative contribution of the fixed variance was smaller in the difficult condition. Therefore, the relationship between interoceptive accuracy and the noise of the primary temporal representation in the difficult condition was not as prominent as in the easy condition. This led to the different results between the conditions. The significant correlation between interoceptive accuracy and temporal precision only under the easy condition was consistent with our assumption.

From the perspective of the pacemaker-accumulator model, the precision in time perception or temporal performance may be explained by the stability of pulse rate of the pacemaker or function of the switch that controls the flow of the pulses to the accumulator. The variability in the pulse rate may fluctuate the number of pulses in physically identical durations, which may result in variability in the subjectively perceived or estimated duration. The delay of the switch at the stimulus onset reduces the number of pulses to be sent to the accumulator, while the delay at the offset increases the number of pulses (Gibbon et al., 1984). Therefore, interoceptive accuracy may be associated with the stability of the pacemaker-accumulator mechanism. This may contribute to constantly generating a more accurate primary temporal representation in internally based timing processing.

Regarding the relationship with vagal activity, two points were inconsistent with the results obtained by Cellini et al. (2015). First is the relationship with temporal accuracy. In the present study, we could not demonstrate a significant correlation between vagal activity and temporal accuracy, as Cellini et al. (2015) did. Our results showed that only *A’* showed a significantly negative correlation with RMSSD under the difficult condition and with the HF component under the easy condition. Observing the insignificant association between *A’* and RMSSD under the easy condition as well as the association between *A’* and HF component under the difficult condition, both tended to be significant (*p* = 0.069, *p* = 0.067, respectively). Considering the sample size, it is possible that these associations did not reach the significant level because of the statistical power. If this is true, the vagal activity may be negatively associated with the sensitivity of the participants, independent of the response criterion. Second is the direction of the correlation. Cellini et al. (2015) showed a positive correlation between vagal activity and temporal ability; a higher HF component was associated with lower errors, and lower sympathetic activity was associated with higher bisection sensitivity. Referring to the neurovisceral integration model by Thayer and Lane (2000, 2009) and Cellini et al. (2015) suggested that higher vagal activity in resting might represent better executive function, such as attention or working memory, which contributed to better performance in the temporal task. However, our results showed that higher vagal activity in resting was associated with lower sensitivity. Fung et al. (2017) also obtained the result that the HF component correlated negatively with temporal accuracy, using a duration reproduction task with supra-second sample duration (from 2 to 15 s). Additionally, Ogden et al. (2019) demonstrated that the perceived duration was shorter and less accurate when parasympathetic activity was increased through paced breathing, using a duration estimation task with sub-second range stimuli (from 200 to 800 milliseconds). Thus, the relationship between vagal activity and temporal ability has demonstrated both positive and negative associations. The negative association may not be explained from the perspective that higher vagal activity indicates better executive function.

Considering that the index of temporal accuracy is defined as the AD between the subjective and real standard, that is, the perceived and real duration, it is clear that vagal activity or interoceptive accuracy can influence temporal accuracy by affecting the perceived duration, not the real duration. Thus, it is necessary to investigate how vagal activity along with interoceptive accuracy or sensitivity explains the perceived duration, which decides the extent of accuracy.

Our multiple linear regression analysis with MSS (perceived duration) as the dependent variable showed two interesting results. First, the interaction effect between vagal activity and interoceptive accuracy was not significant. This implies that vagal activity and interoceptive accuracy may independently exert influence on the perceived duration. Second, the interaction between vagal activity and sensitivity was significant under both the easy and difficult conditions. With higher RMSSD, MSS was shorter in the high *A’* group, but longer in the low *A’* group. The moderation effect of *A’* significantly affected the relationship between RMSSD and MSS in opposite directions, which led to the single insignificant correlation between RMSSD and MSS in the present study. This implies that the relationship between vagal activity and perceived duration could be modified by other factors such as sensitivity. The extent of sensitivity, which differs depending on the tasks or stimuli, may change the relationship between vagal activity and perceived duration, and possibly the relationship between vagal activity and temporal accuracy.

Interestingly, the moderation effect of *A’* was not observed with the HF component, which was the frequency-domain index obtained through the fast Fourier transform, while RMSSD was the time-domain index obtained by calculating the root mean square of the differences in two successive RR intervals, which directly reflected the bodily changes. This might imply that RMSSD played the role of raw materials, that is, bodily changes, for the feeling of duration.

Other multiple regression analyses with DSS (temporal precision) as the dependent variable and with vagal activity and HPS (interoceptive accuracy) as the independent variables with the interaction term were not significant. This model was tested because if participants rely on their heartbeats to estimate time, more variable heart rate (i.e., higher vagal activity) would lead to more variable MSS (mean subjective standard, i.e., higher DSS). Additionally, this association would be more prominent with higher interoceptive accuracy. This assumption was not supported. However, insignificance of the interaction term of vagal activity and interoceptive accuracy supported the possibility that these two factors independently exerted influence on time perception as it was suggested in the analysis of the models with MSS as the depending variables.

Some features relevant to precision in time perception have been revealed. For example, one study showed that higher chronic stress affected the precision in perception of time, not subjective duration (Yao et al., 2015). Other studies showed that the precision in time perception in sub-second range of patients with schizophrenia and bipolar disorder was impaired, while the perceived duration did not differ between these patients and healthy controls (Bolbecker et al., 2014), and the precision of the patients with schizophrenia was impaired regardless of the tasks or stimuli durations, while the impairment of accuracy was lower and task dependent (Thoenes and Oberfeld, 2017). Further, the imprecision in timing was shown to be related to impulsivity in stimulant-dependent human participants (Wittmann et al., 2007), rats (Marshall et al., 2014), and European starlings (Andrews et al., 2021). Regarding the brain region related to precision, it has been reported that a lesion in the medial precentral area of the medial prefrontal cortex and scopolamine injection to this region resulted in impairment of precision in temporal discrimination. However, a lesion in the prelimbic and infralimbic areas of the medial prefrontal cortex did not induce the same effect (Hata and Okaichi, 2004). It is probable that precision in time perception may be a key to explore the modulation of time perception in relation with various mental disorders.

In the present study, we investigated only sub-second range time perception and found the relationship between interoceptive accuracy and temporal precision as well as the moderation effect of participants’ sensitivity on the relationship between vagal activity and perceived duration. In future studies, it is necessary to investigate whether the relationship with precision or sensitivity can be observed in supra-second time, with which the temporal generalization task can be used (Droit-Volet et al., 2001, 2015). Furthermore, we assumed in a very speculative way that the extent of temporal precision might be related with a kind of signal-to-noise ratio, or internal noise (uncertainty) within the individual, which might be related with interoceptive accuracy as well. Smith et al. (2017) updated the neurovisceral integration model introducing the computational perspectives of predictive coding/processing, in which the reliability (or uncertainty) of signals plays an important role in vagal control. Therefore, the relationship between time perception, interoceptive accuracy, and vagal activity might be formulated according to the predictive processing theory. It is worth investigating temporal precision from this perspective in the future.

### Limitations

The present study had two methodological limitations. One is the sample size. The final sample size for the statistical analyses was 29, which might have limited the statistical power of correlation analyses. This may explain the insignificance of some correlations such as interoceptive accuracy and accuracy in time perception. The other limitation is the use of the HCT for the measure of interoceptive accuracy. Although we instructed participants to avoid using prior knowledge to estimate heartbeats, we also told participants that they were allowed to guess. Guessing strategy increases the HPS independently of the real ability to feel interoception (Desmedt et al., 2018). The distribution of the HPS of the present study showed similar characteristics of the result of the task with the original instruction without the explicit inhibition of guessing (Desmedt et al., 2018; Figure 1), which has been criticized for the contribution of non-interoceptive processes (Pennebaker, 1981; Flynn and Clemens, 1988; Ring and Brener, 1996, 2018; Phillips et al., 1999; Windmann et al., 1999; Ring et al., 2015; Desmedt et al., 2018, 2020). The HCT may have other contaminating factors such as the time estimation ability (Desmedt et al., 2020) and response bias or decision threshold (Pohl et al., 2021). Thus, the measure of interoceptive accuracy in the present study may have included various non-interoceptive processes similar to previous studies.

However, contaminating the HCT performance may not have affected the study findings. First, the present study did not detect the contribution of time estimation ability to HCT performance, as the correlation between the counted number of heartbeats and estimated time (MSS) was not significant. This may possibly be because the heartbeats are in supra-second range; meanwhile, we investigated sub-second time perception based on the different process and neural substrates (Rammsayer and Lima, 1991; Grondin et al., 1999; Lewis and Miall, 2003; Wiener et al., 2010; Nani et al., 2019). Thus, the association between the HCT performance and time perception ability in the millisecond range was not due to the involvement of the time estimation ability in the HCT. Second, the multiple linear regression analysis results did not support the assumption that the participants relied on their heartbeats to estimate time. This implies that guessing ability or knowledge of the heart rate may have contributed to better HCT performance but not affected the performance in time perception. Thus, the association between HCT performance and precision or sensitivity in time perception was not due to guessing or heart-rate knowledge. Third, it may be possible that participants with liberal response bias—namely, those who easily reported heartbeats even if they were not sure—might have easily reported the “same” in the temporal generalization task. This tendency may lead to better HCT performance—because more heartbeats are reported—and to less precision in time perception due to more “same” responses to the distractor stimuli. However, this contradicts the results of the present study. Thus, the association between HCT performance and temporal precision is not attributable to response bias.

Numerous factors in the present study may have affected HCT performance, such as the temporal ability in the supra-second range, guessing, heart rate knowledge, or the response criterion. Nevertheless, none of these non-interoceptive processes can be the main factor in the positive association between HCT performance and the temporal precision or sensitivity in the sub-second range. Hence, the present study may have revealed the relationship between precision in sub-second time perception and interoceptive accuracy regardless of the contribution of various non-interoceptive processes, as discussed above.

However, the construct validity of interoceptive accuracy was low in the present study (Schulz et al., 2021). Future studies should confirm the findings of the present study using a novel interoceptive task with more validity (Garfinkel et al., 2022).

## Conclusion

Using the temporal generalization task with sub-second range stimuli, interoceptive accuracy correlated significantly with precision in task performance. Accuracy of mean subjective standard (i.e., accuracy of perceived duration) did not correlate with interoceptive accuracy. The index of sensitivity, which we used from the perspective of signal detection theory, correlated significantly with interoceptive accuracy and vagal activity. Moreover, sensitivity moderated the relationship between vagal activity and perceived duration. The difficulties of the task affected the results possibly for higher influence of the response criterion, which reduced the relative contribution of the fixed variance in temporal precision. Little is known regarding how the interaction between endogenous and exogenous factors, such as autonomic nervous activity, interoceptive accuracy, and task difficulty, modulates time perception. The inconsistency in the results of existing studies may be based on the fact that the unrevealed interaction between endogenous and exogenous factors leads to the moderation effects. The use of indices, such as precision and sensitivity, may help explore new aspects of time perception. In future studies, it is necessary to clarify whether the above-mentioned relationships can be found in the supra-second range as well.

## Data availability statement

The raw data supporting the conclusions of this article will be made available by the authors, without undue reservation.

## Ethics statement

The studies involving human participants were reviewed and approved by the Ethics Committee of Nagoya University. The patients/participants provided their written informed consent to participate in this study.

## Author contributions

MU, VM, and HO designed the study. MU and VM collected and processed the data. MU performed statistical analyses and drafted the manuscript. HO provided the critical feedback. All authors read and approved the final version of the manuscript.
